# Synthesis, Neuroprotection, and Antioxidant Activity of 1,1′-Biphenylnitrones as α-Phenyl-*N-tert*-butylnitrone Analogues in In Vitro Ischemia Models

**DOI:** 10.3390/molecules26041127

**Published:** 2021-02-20

**Authors:** Beatriz Chamorro, David García-Vieira, Daniel Diez-Iriepa, Estíbaliz Garagarza, Mourad Chioua, Dimitra Hadjipavlou-Litina, Francisco López-Muñoz, José Marco-Contelles, María Jesús Oset-Gasque

**Affiliations:** 1 Department of Biochemistry and Molecular Biology, Faculty of Pharmacy, Complutense University of Madrid, Plaza Ramón y Cajal s/n, Ciudad Universitaria, 28040 Madrid, Spain; beatrcha@ucm.es (B.C.); estigara@ucm.es (E.G.); 2 Laboratory of Medicinal Chemistry, Institute of Organic Chemistry (CSIC), Juan de la Cierva 3, 28006 Madrid, Spain; dagarc13@ucm.es (D.G.-V.); daniel.diez@uah.es (D.D.-I.); mchioua@gmail.com (M.C.); 3 Faculty of Health, Camilo José Cela University of Madrid (UCJC), Castillo de Alarcón 49, 28692 Villanueva de la Cañada, Spain; flopez@ucjc.es; 4 Department of Organic Chemistry and Inorganic Chemistry, Alcalá University, 28805 Alcalá de Henares, Spain; 5 Department of Pharmaceutical Chemistry, School of Pharmacy, Faculty of Health Sciences, Aristotle University of Thessaloniki, 54124 Thessaloniki, Greece; hadjipav@pharm.auth.gr; 6 Neuropsychopharmacology Unit, “Hospital 12 de Octubre” Research Institute, Av. de Córdoba s/n, 28041 Madrid, Spain; 7 Instituto Universitario de Investigación en Neuroquímica (IUIN), Universidad Complutense de Madrid. Ciudad Universitaria, 28040 Madrid, Spain

**Keywords:** antioxidants, 1,1′-biphenyl nitrones, free radical scavengers, neuroprotection, oligomycin A/rotenone, oxygen-glucose-deprivation, α-phenyl-*N-tert*-butylnitrone, synthesis

## Abstract

Herein, we report the neuroprotective and antioxidant activity of 1,1′-biphenyl nitrones (**BPNs**) **1**–**5** as α-phenyl-*N*-*tert*-butylnitrone analogues prepared from commercially available [1,1′-biphenyl]-4-carbaldehyde and [1,1′-biphenyl]-4,4′-dicarbaldehyde. The neuroprotection of **BPNs** **1**-**5** has been measured against oligomycin A/rotenone and in an oxygen–glucose deprivation in vitro ischemia model in human neuroblastoma SH-SY5Y cells. Our results indicate that **BPNs 1**–**5** have better neuroprotective and antioxidant properties than α-phenyl-*N*-*tert*-butylnitrone (**PBN**), and they are quite similar to *N*-acetyl-L-cysteine (**NAC**), which is a well-known antioxidant agent. Among the nitrones studied, homo-bis-nitrone **BPHBN5**, bearing two *N*-*tert*-Bu radicals at the nitrone motif, has the best neuroprotective capacity (EC_50_ = 13.16 ± 1.65 and 25.5 ± 3.93 μM, against the reduction in metabolic activity induced by respiratory chain blockers and oxygen–glucose deprivation in an in vitro ischemia model, respectively) as well as anti-necrotic, anti-apoptotic, and antioxidant activities (EC_50_ = 11.2 ± 3.94 μM), which were measured by its capacity to reduce superoxide production in human neuroblastoma SH-SY5Y cell cultures, followed by mononitrone **BPMN3**, with one *N*-Bn radical, and **BPMN2**, with only one *N*-*tert*-Bu substituent. The antioxidant activity of **BPNs** **1**-**5** has also been analyzed for their capacity to scavenge hydroxyl free radicals (82% at 100 μM), lipoxygenase inhibition, and the inhibition of lipid peroxidation (68% at 100 μM). Results showed that although the number of nitrone groups improves the neuroprotection profile of these **BPNs**, the final effect is also dependent on the substitutent that is being incorporated. Thus, **BPNs** bearing *N*-*tert*-Bu and *N*-Bn groups show better neuroprotective and antioxidant properties than those substituted with Me. All these results led us to propose homo-*bis*-nitrone **BPHBN5** as the most balanced and interesting nitrone based on its neuroprotective capacity in different neuronal models of oxidative stress and in vitro ischemia as well as its antioxidant activity.

## 1. Introduction 

The Reactive Oxygen Species (ROS) formed in the metabolism in living organisms play key roles acting at low to moderate concentrations in many important cell processes; however, at high concentrations, they produce negative effects in key physiological biomolecules, such as DNA, proteins, and lipids [1]. As a result, oxidative stress (OS) is involved in diverse pathological conditions [2]. In particular, in cerebral ischemia (CI), OS is one of the most important molecular factors involved in its progression and development [3], concentrating most of the therapeutic approaches and strategies to find a potential cure. 

**Figure 1:**
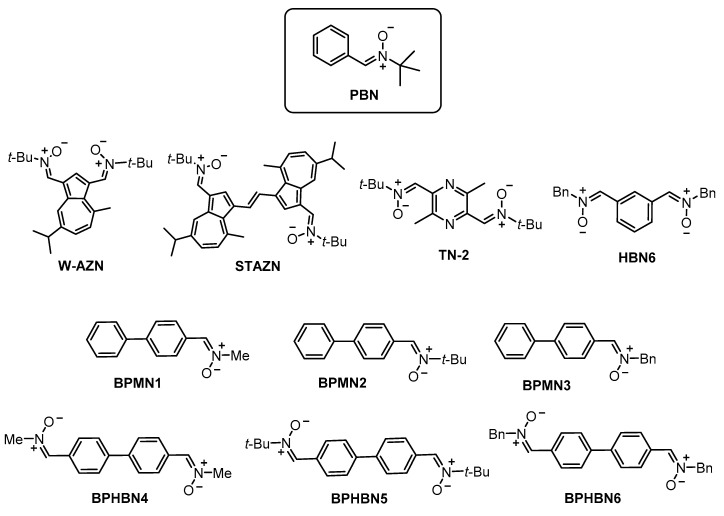
Structures of α-phenyl-*N*-*tert*-butylnitrone (**PBN**), *bis*-nitrones **W-AZN**, **STAZN**, **TN-2**, **HBN6,** and the 1,1′-biphenyl nitrones (**BPNs**) **1-6** described in this work.

Thus, in the search for new scavenging ROS [4], nitrones, well-known organic molecules typically used as radical traps, appear as suitable drugs to treat CI [5]. The first proposed nitrone for stroke was α-phenyl-*N*-*tert*-butylnitrone (**PBN**) (Figure 1), which prevented and reversed traumatic shock injury in rats [6]. **PBN** was the starting point of a number of new nitrones targeted for the therapy of CI, such as **NXY-059**, which was the first nitrone submitted to clinical trials, although it failed in advanced phase III, as no significant effect was observed when compared with placebo [7]. In spite of this, neuroprotective and antioxidant nitrones [8] are still investigated for the potential treatment of CI in a number of laboratories. Thus, for instance, in our current research targeted to identify new antioxidant nitrones for the potential stroke therapy [9], we have reported nitrones derived from (hetero)aromatic aldehydes [10,11], as well as quinolylnitrones [12,13,14,15], cholesteronitrones [16], and indanonitrones [17] derived from **PBN**.

Bis-nitrones form also a small group of antioxidant agents, showing potent neuroprotective properties. This is the case of *bis*-nitrone **W-AZN** (Figure 1), which is an azulenyl spin trap possessing neuroprotective effects in an animal model of cerebral ischemia, and it is able also to attenuate the in vivo MPTP neurotoxicity [18], or **STAZN** (Figure 1), which is a second-generation of potent antioxidant azulenyl nitrone [19], whose neuroprotective capacity has been confirmed in focal ischemia models [20]. Bis-nitrone **TN-2** (Figure 1) bears two *tert*-butyl nitrone motifs anchored to a pyrazine as the heterocyclic ring core, showing high neuroprotective effect in in vitro and in vivo models of stroke, as a very plausible consequence of its ability to trap ROS HO^●^ and O_2_^●-^ [21]. In this context, we have recently identified (1*Z*,1′*Z*)-1,1′-(1,3-phenylene)bis(*N*-benzylmethanimine oxide) (**HBN6**) (Figure 1) as a potent neuroprotective ligand overcoming the neuroprotective and antioxidant capacities of **PBN** [22].

With these precedents in mind, and continuing with our efforts in this area, in this work, we report the synthesis, neuroprotective, and antioxidant activity of 1,1′-biphenyl nitrones (**BPNs**), as **PBN** analogues derived from [1,1′-biphenyl]-4-carbaldehyde, such as [1,1′-biphenyl]-4-carbaldehyde mononitrones (**BPMNs**) **1**–**3**, and from [1,1′-biphenyl]-4,4′-dicarbaldehyde, such as [1,1′-biphenyl]-4,4′-dicarbaldehyde homo-*bis*-nitrones (**BPHBNs**) **4**–**6** (Figure 1). As a result of this work, we have identified **BPHBN5** as the most balanced nitrone based on its neuroprotective capacity in different neuronal models of oxidative stress, an in vitro ischemia model, and antioxidant assays. 

## 2. Results and Discussion

### 2.1. Chemistry

**Scheme 1:**
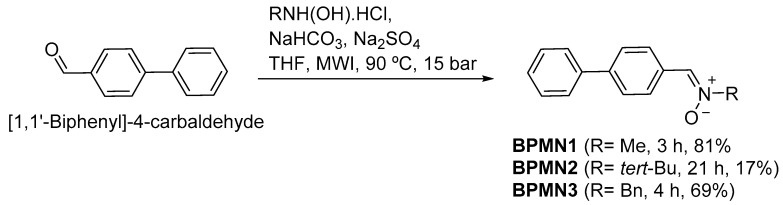
Synthesis of BPMNs 1-3.

**Scheme 2:**
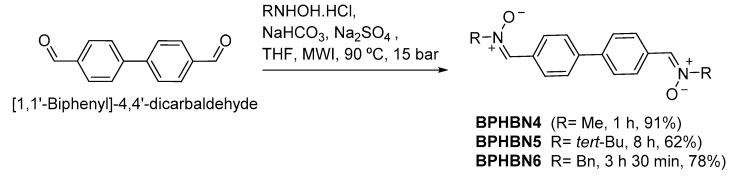
Synthesis of [1,1′-biphenyl]-4,4′-dicarbaldehyde homo-*bis*-nitrones (BPHBNs) 4–6.

The synthesis of **BPMNs 1**-**3** has been carried out as shown in Scheme 1, starting from commercial and readily available compounds derived from commercially available [1,1′-biphenyl]-4-carbaldehyde, and the selected and appropriate *N*-alkyl hydroxylamine hydrochloride, in THF, in the presence of sodium bicarbonate and sodium sulfate, by irradiation (90 °C, 250 W, 15 bar) as shown in Scheme 1. Similarly, but starting from [1,1′-biphenyl]-4,4′-dicarbaldehyde, **BPHBNs 4**–**6** were obtained in good yields (Scheme 2). 

All new or known compounds have been isolated as pure *Z* isomers at the double PhC=N(O)R bond, as usual, and they gave analytical and spectroscopic data in good agreement with their structures as well as with the previously described data in the literature for known nitrones **BPMN1** [23], **BPMN2** [24], and **BPHBN4** [23], but these studies were not related to their neuroprotective and antioxidant properties. Unfortunately, **BPHBN6** was too insoluble to be analyzed in the biological tests, and consequently, the neuroprotective profile of this compound has not been assayed.

### 2.2. Neuroprotection Studies of **BPNs 1**–**5**

#### 2.2.1. Neuroprotection Analysis in an Oligomycin A/Rotenone (OR) Model

One of the first events taking place in the initial stages of stroke is the collapse of the mitochondrial electron transport chain (ETC), which leads to extended cell death and brain damage due to the formation of ROS. In order to mimic this event into suitable experiments, we tested the effect of the BPNs on cell death induced by oligomycin A and rotenone (O/R), which are inhibitors of mitochondrial complexes V and I, respectively. To this end, we used the XTT cell viability test and a colorimetric assay that detects the cellular metabolic activities. Based on a previous work from our laboratory [22], we selected the appropriate experimental conditions and tested the neuroprotective effect of BPNs 1-5 at different concentrations (0.1–1000 μM), which were added 10 min before the administration of O10 μM /R30 μM (O/R), and using **PBN** and **NAC** as reference compounds, at the same concentrations (0.1–1000 μM), as reference compounds [22,25].

**Figure 2:**
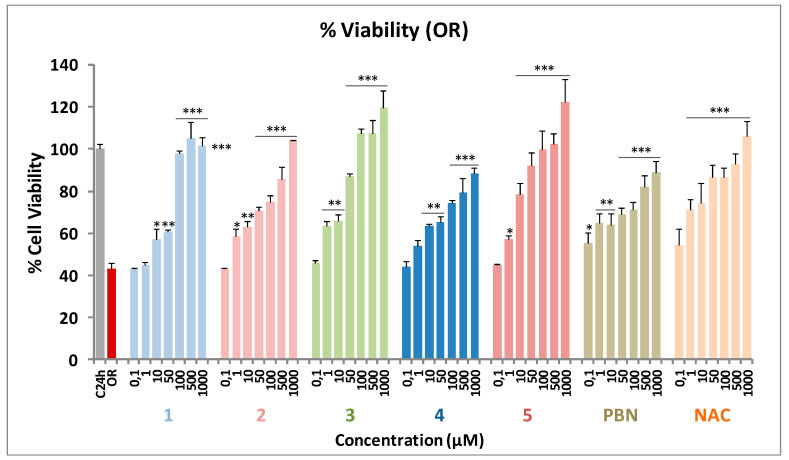
Neuroprotective effect of BPNs 1-5, PBN, and *N*-acetyl-L-cysteine (NAC) on SH-SY5Y human neuroblastoma metabolic activity after treatment with oligomycin A 10 μM/rotenone 30 μM (O/R). Bars show the percentage of cell viability after treatment with O/R, with, or without, BPNs 1-5, PBN, and NAC, at the indicated concentrations. Values are the mean ± SEM of three experiments, each one performed in triplicate (n = 3). The statistics compare differences with the OR condition at * *p* < 0.05, ** *p*< 0.01 and *** *p* < 0.001 (one-way ANOVA, followed by Holm−Sidak analysis as a test post hoc.

As shown in Figure 2, a 57% inhibition of neuroblastoma cells viability [43.23 ± 2.61% cell viability (mean ± SEM)] was observed upon treatment with O10/R30 for 24 h. This effect was reverted after incubation with **BPNs 1-5**, **PBN**, and **NAC** for 24 h in a concentration-dependent manner (Figure 2). The neuroprotection study, considering 100% neuroprotection as the difference between C24 h viability (100 ± 2.55%; mean ± SEM; n = 20) and OR (43.23 ± 2.61; mean ± SEM; n = 16) revealed that the most potent nitrone was **BPHBN5**. 

**Figure 3:**
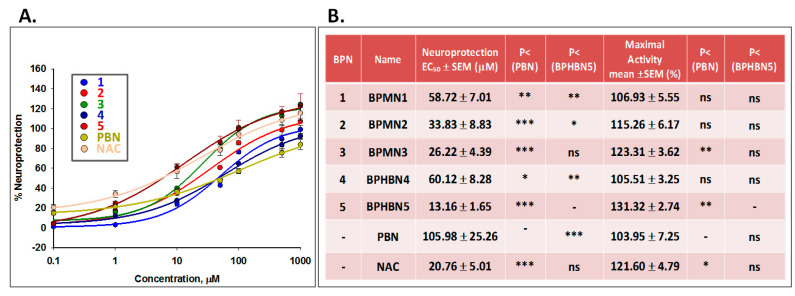
Neuroprotective effects of BPNs 1-5, PBN, and NAC against cell death induced by oligomycin A 10 μM/rotenone 30 μM (OR) treatment in SHSY5Y human neuroblastoma cells. (**A**) Dose–response curves showing the percentage of neuroprotection of different compounds at the indicated concentrations. The curve adjustments to estimate the EC_50_ were carried out by non-linear ponderated regression analysis of minimal squared, using logistic curves f1 = min + (max-min) / (1+(x/EC_50_) ^ (-Hillslope). Data represent the mean ± SEM of three experiments, each one done in triplicate (n = 9). The analysis was implemented using the software SigmaPlot v.11. (**B**) EC_50_ and maximal activity values for the indicated compounds. The statistics compare the differences between EC_50_ or maximal activity values for different compounds tested against PBN or BPMN2 at * *p* < 0.05, ** *p* < 0.01, and *** *p* < 0.001 (ANOVA one way).

Figure 3 gathers the analyses of concentration–response curves for **BPNs 1**-**5**, compared with **PBN** and **NAC**, in the range of 0.1 μM to 1 mM (Figure 3A) and the corresponding EC_50_ values, and the highest neuroprotective activities (Figure 3B). EC_50_ values, from the lowest to the highest, follows the order: **BPHBN5** **≤ NAC** **≤** **BPMN3** **≤** **BPMN2** **≤** **BPMN1** **≤** **BPHBN4 << PBN**. These results indicate that tested **BPNs 1**–**5** have a more potent neuroprotective effect than **PBN** on cell death induced by respiratory chain blockers. Regarding the maximal activity, only **BPMN3**, bearing one *N*-benzyl radical, and **BPHBN5**, bearing two *N*-*tert*-butyl radicals, had a greater activity than **PBN**, indicating that these two nitrones are the most potent neuroprotective **BPNs** in this model of neuronal damage. The high neuroprotection observed for nitrones **2**, **3**, and **5** exceeds that of the parent **PBN** and is very similar to that of **NAC**. Thus, from the structure–activity relationship (SAR) point of view, it should be noted that among these **BPNs**, those bearing *N*-benzyl or *N*-*tert*-butyl groups at the nitrogen atom of the nitrone motif systematically afforded a higher neuroprotection.

#### 2.2.2. Neuroprotection Analysis in an Oxygen and Glucose Deprivation (OGD) followed by Oxygen and Glucose Resupply (IR) Model

**Figure 4:**
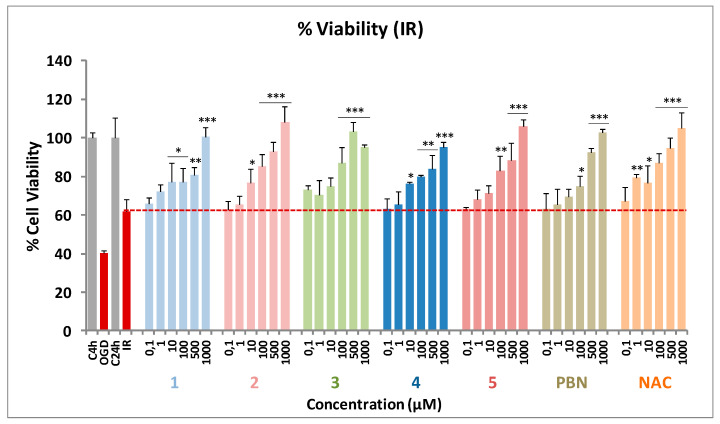
Neuroprotective effect of BPNs 1–5, PBN, and NAC on SH-SY5Y human neuroblastoma metabolic activity after oxygen glucose deprivation (4 h) and ischemic reperfusion (24 h) (I/R). Bars show the percentage of cell viability at the indicated concentrations, BPNs 1–5, PBN, and NAC at the indicated concentrations. Values are the mean ± SEM of three experiments, each one performed in triplicate (n= 3). The statistics compares differences with IR condition alone (red dotted line) at * *p* <0.05, ** *p* <0.01, and *** *p* < 0.001 (one-way ANOVA, followed by Holm−Sidak analysis as a test post hoc.

Next, the neuroprotective effect of BPNs 1–5 was evaluated in an in vitro oxygen glucose deprivation (OGD) model, followed by oxygen and glucose resupply (R), and an in vitro model of ischemia reperfusion (IR) [22,26]. Tested compound concentrations ranged from 0.01 to 1000 μM, after IR. After OGD (I) (4 h), a loss of metabolic activity of about 60% (40.07 ± 2.37% cell viability, mean ± SEM; n = 16) was observed, showing a small cell recovery after 24 h reperfusion (IR) of about a 20–25% (61.96 ± 8.56% cell viability; mean ± SEM; n = 16). BPNs 1–5 were able to partially or even totally reverse the cell loss of metabolic activity induced by IR in a concentration-dependent manner (Figure 4). These data revealed that BPNs 2, 3, and 5 were the most potent nitrones. Among BPMNs 1-3, BPMNs 2 and 3 provided the better neuroprotection, regardless of the dose, whereas among BPHBNs, the best neuroprotective was BPHBN5. To sum up, in the OGD experiment, nitrones 2, 3, and 5 showed the best ability to counteract the IR-induced decrease in metabolic capacity in human neuroblastoma cells, which is in good agreement with the results observed with the inhibitors of the mitochondrial ETC. 

**Figure 5:**
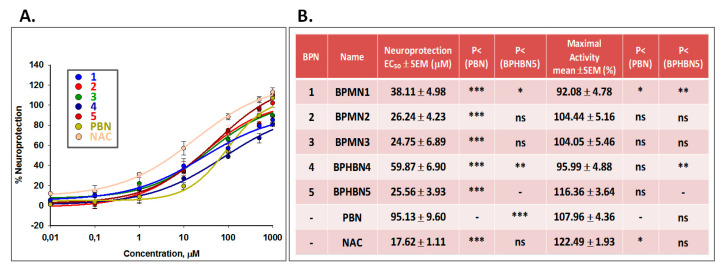
Neuroprotective effects of BPNs 1-5, PBN, and NAC against cell death induced by I/R treatment in SHSY5Y human neuroblastoma cells. (**A**) Dose–response curves showing the percentage of neuroprotection of different compounds at the indicated concentrations. The curve adjustments to estimate the EC_50_ were carried out by non-linear ponderated regression analysis of minimal squared, using logistic curves f1 = min+(max-min) /(1+(x/EC_50_)^(-Hillslope). Data represent the mean ± SEM of three experiments, each one done in triplicate (n= 3). The analysis was implemented using the software SigmaPlot v.11. (**B**) EC_50_ and Maximal Activity values for the indicated compounds. The statistics compares the differences between EC_50_ or maximal activity values for different compounds tested against PBN or BPMN2 at * *p* < 0.05, ** *p* < 0.01, and *** *p* < 0.001 (one-way ANOVA).

Figure 5 gathers the analyses of concentration–response curves for **BPNs 1-5,** compared with **PBN** and **NAC**, in the range of 0.1 μM to 1 mM (Figure 5A) and the corresponding EC_50_ values, and the highest neuroprotective activities (Figure 5B). As shown in Figure 5B, the EC_50_ values, from the lowest to the highest neuroprotective nitrone follows the order: **NAC** ≤ **BPMN3** ≤ **BPHBN5** ≤ **BPMN2** < **BPMN1** < **BPHBN4** << **PBN**. However, there were no significant differences in the maximal neuroprotective activity determined for all these compounds. Thus, from the SAR point of view, note that (1) the best neuroprotective nitrones **2**, **3**, and **5** bear either *N-tert*-Bu (**BPMN2** and **BPHBN5**) or *N*-Bn (**BPMN3**) groups at the nitrogen atom of the nitrone motif; (2) there are no significant differences in neuroprotective capacity between nitrones carrying one (**BPCMN2**) or two *tert*-Bu radicals (**BPHBN5**); (3) the worst neuroprotective capacity is shown by nitrones **1** and **4**, bearing *N*-Me as substituent, and this capacity is worsened by adding a second *N*-Me group, as **BPHBN4** is worse than **BPMN1**; and (4) the neuroprotection afforded by **BPNs 1**–**5** is better that the induced by **PBN**, and the neuroprotective capacity of the three best neuroprotective nitrones is very similar to that of **NAC** (EC_50_ = 17.62 ± 1.93 μM).

#### 2.2.3. Effect of **BPNs 1–5** on Necrotic and Apoptotic Cell Death Induced by Oxygen and Glucose Deprivation followed by Oxygen and Glucose Resupply (IR) Model

During an ischemic stroke, there is massive cell death due to necrosis, and, as a consequence, the plasma membrane is broken or significantly permeabilized [27]. Under these circumstances, lactate dehydrogenase (LDH), a soluble cytosolic enzyme, easily crosses the damaged membrane, and for this reason, it is possible to determine the extent of the cell necrosis taking place in the OGD experiment by comparing its extracellular to its intracellular activity. 

**Figure 6:**
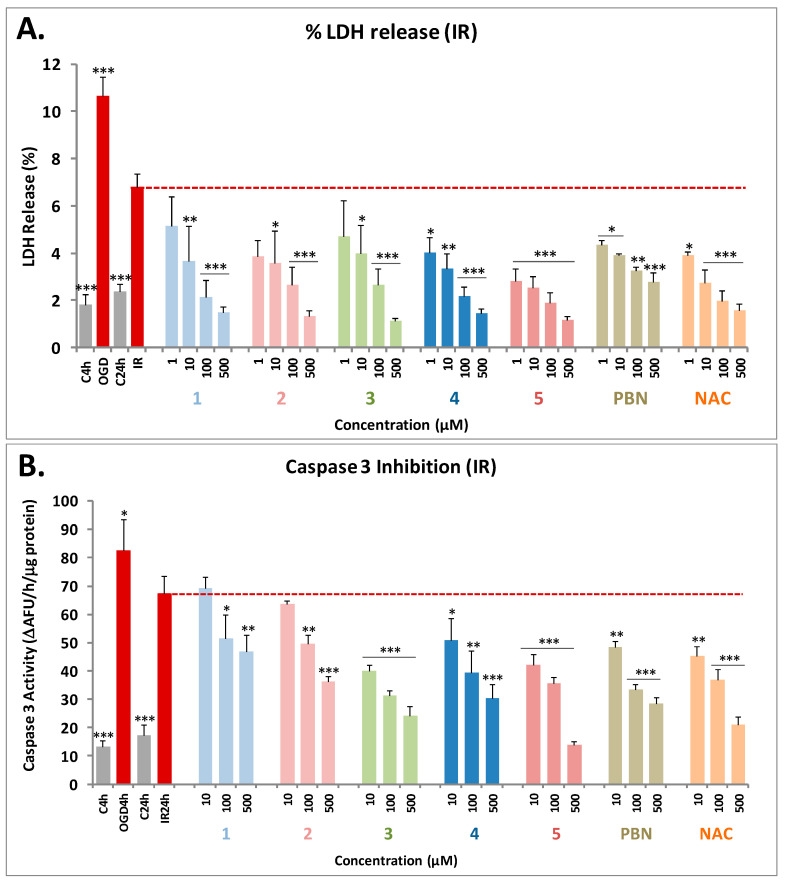
Neuroprotective effects of PBNs 1–5, PBN, and NAC against necrotic (**A**) and apoptotic (**B**) cell death induced by IR treatment in SHSY5Y human neuroblastoma cells. A) Bars show the percentage of lactate dehydrogenase (LDH) release after oxygen and glucose deprivation (OGD) (4 h) and I/R (24 h), without treatment (IR 24 h) or treated with PBNs 1–5, PBN and NAC, at the indicated concentrations. B) Bars show caspase 3 activity expressed as ΔAFU/μg protein/min after OGD (4 h) and I/R (24 h) alone or treated with PBNs 1–5, PBN, and NAC, at the indicated concentrations. (A-B) Values are the mean ± SEM of three experiments, each one performed in triplicate (n = 3), and we compare the effect of different treatments or compounds on the I/R (24h) condition alone, that is in the absence of these compounds. Data were statistically analyzed by one-way ANOVA, followed by Holm–Sidak as test post hoc. * *p* < 0.05; ** *p* < 0.01; and *** *p* < 0.001. UAF = Arbitrary Fluorescent Units.

As shown in Figure 6A, from the values obtained from the measurement of the LDH release after OGD for 4 h, followed by 24 h ischemic reperfusion (I/R) on neuroblastoma cells, by adding **BPNs 1**–**5** at 1–500 μM concentrations, and **PBN** and **NAC**, we concluded that all the **BPNs** significantly decreased the release of LDH in a concentration-dependent manner, reaching 100% of maximal inhibition of LDH release at concentrations between 100 and 500 μM (Figure 6A). The nitrones that showed that the greatest anti-necrotic activity was **BPHBN5**, bearing two *N-tert*-Bu radicals as substituents and very similar to that achieved by **NAC**. The remaining nitrones bearing *N-*Bn (**BPMN3**) and *N*-Me (**BPMN1** and **BPHBN4**) substituents also showed a good anti-necrotic profile, although it was lower than that **BPHBN5** and **BPHBN2**. Once again, **PBN** showed the lowest anti-necrotic activity among all the nitrones tested.

Next, and in order to evaluate the extent of cell death by apoptosis, we determined the caspase-3 activity by using DEVD-AMC as a substrate, which affords fluorescent AMC upon hydrolysis. So, after OGD (4 h), and adding **BPNs 1**–**5**, **PBN**, and **NAC**, at 10–500 μM concentrations, followed by IR (24 h), the cells were lysated, DEVD-AMC was added, and the fluorescence was measured. 

As shown in Figure 6B, we concluded that in general, all the tested **BPNs** had a concentration-dependent antiapoptotic effect. However, in general, they protect less efficiently from the apoptotic than from necrotic cell death. The most potent was **BPHBN5**, which at concentrations of 500 μM reached 100% inhibition, as did **NAC**. The antiapoptotic capacity of **BPMN3** was also very good. In general, there is a clear SAR between the structure and the antiapoptotic effect, since nitrones bearing two *N-*Me (**BPHBN4**) or *N-tert*-Bu (**BPHBN5**) groups, and especially the last one, showed greater effect than their corresponding nitrone counterparts bearing one *N-*Me (**BPMN1)** or *N-tert*-Bu (**BPMN2)** group.

In conclusion, nitrones **5** and **3** have the best anti-necrotic and anti-apoptotic effects, similar to **NAC** and better than **PBN**, supporting their effects on neuroprotective activity on the neuronal metabolic activity described above. 

### 2.3. Basal Neurotoxicity of BPNs 1–5, PBN, and NAC

**Figure 7:**
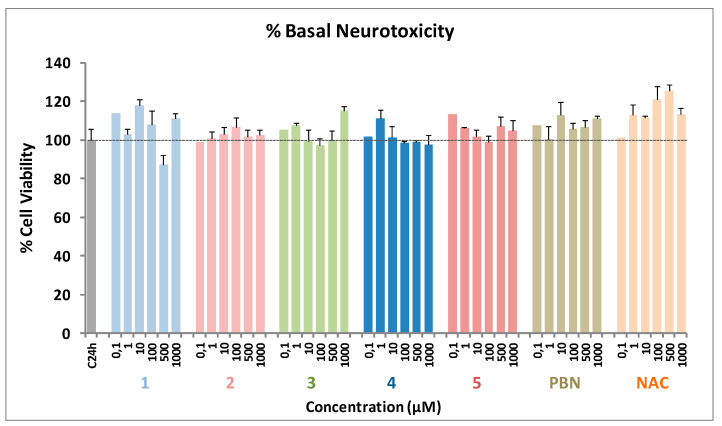
**Effect ofBPNs1–5, PBN, and NAC on human neuroblastoma SH-SY5Y cell viability under basal conditions.** Bars represent the percentage of cell viability in the presence of the compounds at the indicated concentrations. Cell viability for the untreated cells (C24h) was assigned 100% (100 ± 5.52%). Values are the mean ± SEM of five experiments, each one in triplicate (n = 5). Statistics was performed by one-way ANOVA test. There were no significant differences with respect to control. Analysis of results above 100% is not shown.

Based on the results described above, the analysis of the possible neurotoxicity of **BPNs 1–5** was mandatory, and carried out by measuring the cell viability with XTT, but without adding any toxic insult. As shown in Figure 7, **PBNs 1–5**, as well as **PBN** and **NAC**, did not show any neurotoxic effects at the basal level.

### 2.4. Antioxidant Capacity of **BPNs 1–5, PBN,** and **NAC**: Production and Scavenging of Superoxide Radical in Human Neuroblastoma SH‑SY5Y Cells

The results shown in the previous sections prompted us to investigate whether the observed neuroprotection was a consequence of their capacity to act as antioxidants and ROS scavengers, particularly of superoxide radical anion (O_2_^●^). O_2_^●^ detection was carried out by using dihydroethidium (DHE), after OGD (3 h) and IR (3 h), with or without **BPNs** **1–5**, including **PBN** and **NAC**. Compound concentrations from 0.1 to 1000 μM were tested, after I/R. 

**Figure 8:**
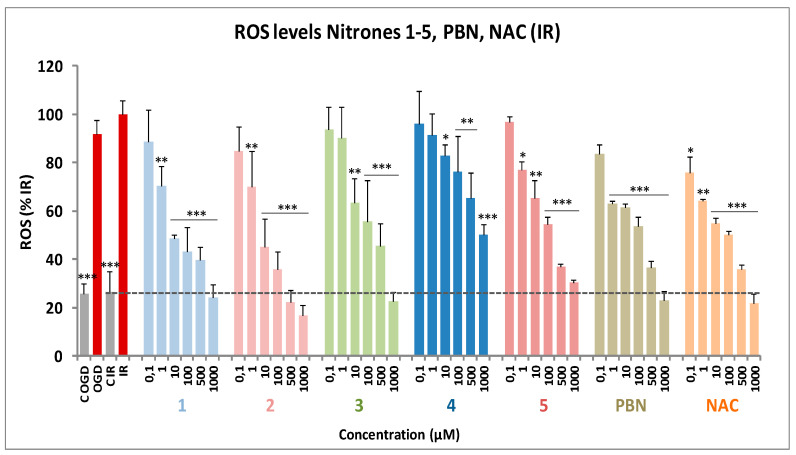
Inhibitory effects of BPNs 1-5, PBN, and NAC on Reactive Oxygen Species (ROS) (superoxide) production in SHSY5Y human neuroblastoma cell cultures exposed to OGD (4 h) and 3 h reperfusion (I/R). Bars show the percentage of ROS formed after OGD and IR with, or without, BPNs 1-5 or PBN and NAC, at the indicated concentrations. Values are mean ± SEM of at least three experiments, each one performed in triplicate (n = 3). Values for ROS in basal conditions were calculated as 0.46 ± 0.08 UAF/min/100.000 cells (n = 4). The statistics compares the effect of I/R against controls or the effect of the different compounds respect to I/R at * *p* < 0.05, ** *p* < 0.01, *** *p* < 0.001 (one-way ANOVA followed by Holm–Sidak analysis post hoc).

As shown in Figure 8, the ROS production level after IR (0.459 ± 0.080 UAF/min/100.000 cells; mean ± SEM; n = 12) was higher but non-significantly different (ns, one-way Anova test) than ROS production after OGD alone (0.422 ± 0.078 UAF/min/100.000 cells mean ± SEM; n = 12). As expected, **BPNs** **1-5** were able to partially or totally reverse the increase in ROS levels induced by I/R in a concentration-dependent manner (Figure 8). 

**Figure 9:**
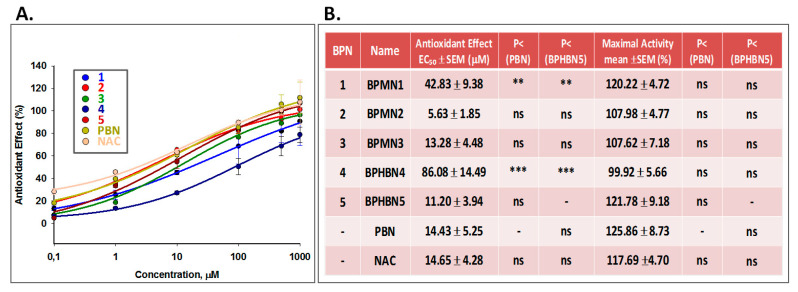
**Antioxidant effect of BPNs 1–5, PBN, and NAC after I/R in human neuroblastoma SH-SY5Y cells. (A)** Dose–response curves showing the percentage of antioxidant effect of different compounds at the indicated concentrations. The curve adjustments to estimate the EC_50_ were carried out by non-linear ponderated regression analysis of minimal squared, using logistic curves f1 = min+(max-min) /(1+(x/EC_50_)^(-Hillslope). Data represent mean ± SEM of three experiments, each one done in triplicate (n = 3). The analysis was implemented using the software SigmaPlot v.11. **(B)** EC_50_ values and maximal antioxidant activities for the indicated compounds. The statistics compares the differences between EC_50_ or maximal activities values for different compounds tested against **PBN** or **BPMN2** at * *p* < 0.05, ** *p* < 0.01 y *** *p* < 0.001 (one-way ANOVA, followed by Holm–Sidak analysis as a post hoc test).

The analyses of concentration–response curves data (Figure 9A) and calculations of EC_50_ and the highest antioxidant activities for **BPNs 1**–**5**, **PBN**, and **NAC** are shown in Figure 9B. The EC_50_ values, from the lowest to the highest, follow the order: **BPMN2** < **BPHBN5** ≤ **BPMN3** ≤ **PBN** ≤ **NAC** << **BPMN1** << **BPMN4**. As the highest neuroprotective activity (maximal activities) was similar for all compounds tested, we conclude that regarding the antioxidant capacity against IR-induced superoxide production, nitrones **2**, **5**, and **3** exhibit the best antioxidant properties, which is very similar to that of **PBN** and **NAC**. In summary, and from the SAR point of view, once again, **BPMN2** and **BPHBN5**, bearing *N-tert*-Bu, and **BPMN3** bearing *N-*Bn substituents, were the most potent nitrones of the entire series. Therefore, these data confirm again that nitrones substituted with *tert*-Bu and Bn groups are the best antioxidants as well as neuroprotective nitrones, although in this case, and unlike their neuroprotective capacity, the fact of having two nitrone groups does not seem to increase their antioxidant effects as ROS scavengers; rather, it decreases them. 

Based on the antioxidant results shown in Section 2.4, we decided to carry out additional antioxidant assays in order to better analyze the antioxidant potential of nitrones **1–5**, such as the decolorizing 2,2′-azino-bis(3-ethylbenzthiazoline-6-sulfonic acid) (ABTS) test, the scavenging of hydroxyl free radicals, the lipoxygenase (LOX) inhibition, and the inhibition of lipid peroxidation (ILPO). **PBN**, nordihydroguaiaretic acid (NDGA), and Trolox were used as standards for comparative purposes.

As shown in Table 1, the ability of **BPNs 1**–**5** to trap ABTS^+•^ species was very poor, while their ability to scavenge hydroxyl radicals was remarkable, showing 82–93% values in the same range as Trolox. In overall, mono nitrone **BPMN3**, bearing an *N*-Bn group at the nitrone moiety was the most balanced one, scavenging 84% hydroxyl free radicals, inhibiting with an IC_50_ value of 57.5 µM LOX, and inhibiting 100% LPO activity, comparing quite well with the reference standards used for comparison purposes followed by homo-*bis*-nitrone **BPHBN5**, bearing two *N*-*tert*-Bu groups at the nitrone moieties, showing 82% scavenging of hydroxyl free radicals, inhibiting with an IC_50_ value of 100 µM LOX, and inhibiting 68% LPO. 

## 3. Materials and Methods

### 3.1. Chemistry

**General Methods.** Compound purification was performed by column chromatography with Merck Silica Gel (40-63 µm) or by flash chromatography (Biotage Isolera One equipment) and the adequate eluent for each case. Reaction course was monitored by thin layer cromatography (t.l.c.), revealing with UV light (λ= 254 nm) and ethanolic solution of vanillin or ninhydrin. Melting points were determined using a Reichert Thermo Galen Kofler block and are uncorrected. Samples were dissolved in CDCl_3_ or DMSO-*d_6_* using TMS as an internal standard for ^1^H NMR spectra. In ^13^C NMR spectra, CDCl_3_ central signal (77.0 ppm) and DMSO-*d* (39.5 ppm) were used as references. ^1^H NMR and ^13^C NMR spectra were obtained in Bruker Avance 300 (300 MHz) and Bruker Avance 400 III HD (400 Hz) spectrometers. Chemical shifts (δ) are given in ppm. Coupling constants (*J*) are given in Hz. Signal multiplicity is abbreviated as singlet (s), doublet (d), triplet (t), quartet (c), doublet of doublets (dd), triplet of doublets (td), or multiplet (m). Values with * can be interchanged. IR spectra were recorded on a Perkin-Elmer Spectrum One B spectrometer. Units are cm^-1^. Low-resolution mass spectra were recorded on an Agilent HP 1100 LC/MS Spectrometer, whereas high-resolution mass spectrometry (Exact Mass) was performed in an AGILENT 6520 Accurate-Mass QTOF LC/MS Spectrometer. Elemental analysis was performed in an Elementary Chemical Analyzer LECO CHNS-932. The following numeration has been used in the nitrones NMR assignments:


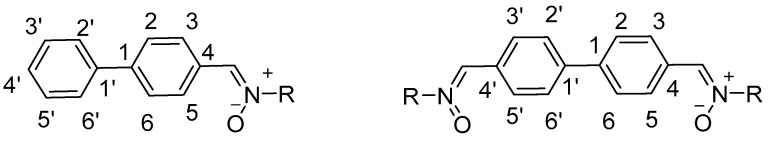


**General method for the synthesis of nitrones 1-3**. To a solution of [1,1′-biphenyl]-4-carbaldehyde (1 equiv), in dry THF (0.05M), Na_2_SO_4_ (3 equiv), anhydrous NaHCO_3_ (3 equiv), and the appropriate hydroxylamine hydrochloride (1.2 equiv) were added, and the mixture was irradiated (90 ºC/ 15 bar) and stirred for the indicated time in each case. When the reaction was complete (TLC analysis), the solvent was removed, and the crude was purified by flash-chromatography. 

**(Z)-1-([1,1′-Biphenyl]-4-yl)-N-methylmethanimine oxide (BPMN1)** [23]. Following the **General method**, [1,1′-biphenyl]-4-carbaldehyde (91 mg, 0.5 mmol) was treated with NaHCO_3_ (126 mg, 1.5 mmol), Na_2_SO_4_ (213 mg, 1.5 mmol), and MeNHOH.HCl (50.1 mg, 0.6 mmol), in THF (20 mL), and irradiated for 3 h, to give, after purification (AcOEt:MeOH, 9:1, v/v), nitrone **BPMN1** (86.7 mg, 81%), as a white solid: mp 134-6 ºC; IR (KBr) ν 3392, 1406, 1155 cm^−1^; ^1^H NMR (400 MHz, CDCl_3_) δ 8.30 (d, *J*= 8.5 Hz, 2H, H3, H5), 7.65 (dd, *J*= 8.4, 2.7 Hz, 2H, H2, H6), 7.64 (d, *J*= 8.5 Hz, 2H, H2’, H6’), 7.46 (t, *J*= 7.5 Hz, 2H, H3’, H5’), 7.41 [(s, 1H, HC=N(O)], 7.40 (t, *J*= 7.1 Hz, 1H, H4’), 3.91 (s, 3H, CH_3_); ^13^C NMR (101 MHz, CDCl_3_) δ 143.0 (1C, C1), 140.2 (1C, C1´), 134.9 [1C, HC=N(O)], 129.5 (1C, C4), 129.0 (4C, C3/C5, C3’/5’), 127.9 (1C, C4’), 127.1 (4C, C2/C6/C2´/C6´), 54.5 (CH_3_); MS (ESI) *m/z*: 212 [M+1]^+^, 234 [M+Na]^+^, 445 [2M+Na]^+^. Anal. Calcd. for C_14_H_13_NO.2/7 H_2_O: C, 77.70; H, 6.32; N, 6.47. Found: C, 77.72; H, 6.14; N, 6.52.

**(*Z*)-1-([1,1′-Biphenyl]-4-yl)-*N*-(*tert*-butyl)methanimine oxide (BPMN2) [24].** Following the **General method**, [1,1′-biphenyl]-4-carbaldehyde (91 mg, 0.5 mmol) was treated with NaHCO_3_ (126 mg, 1.5 mmol), Na_2_SO_4_ (213 mg, 1.5 mmol) and *tert*-BuNHOH.HCl (75.4 mg, 0.6 mmol), in THF (20 mL), and irradiated for 21 h to give, after purification (hex:AcOEt, 3:2, v/v), nitrone **BPMN2** (21.4 mg, 17%), as a white solid: mp 109-111 ºC; IR (KBr) ν 3434, 1130, 1122 cm^-1^; ^1^H NMR (400 MHz, CDCl_3_) δ 8.38 (d, *J*= 8.4 Hz, 2H, H3, H5), 7.65 (d *J*= 8.4 Hz, 2H, H2, H6), 7.64 (tt *J*= 8.6, 1.7 Hz, 2H, H2’, H6’), 7.60 [s, 1H, HC=N(O)], 7.46 (tt, *J*= 7.8, 1.3 Hz, 2H, H3’, H5’), 7.38 (tt, *J*= 7.4, 2.1 Hz, 1H, H4’), 1.67 [s, 9H, C(CH_3_)_3_]; ^13^C NMR (101 MHz, CDCl_3_) δ 142.6 (1C, C1), 140.4 (1C, C1´), 130.1 (1C, C4), 129.6 [1C, HC=N(O)], 129.3 (2C, C3/C5), 128.9 (2C, C3´/C5´), 127.8 (1C, C4´), 127.2 (2C, C2/C6)*, 127. 1 (2C, C2´/C6´)*, 70.9 [N(O)*C*(CH_3_)_3_], 28.5 [3C, C(*C*H_3_)_3_]; MS (ESI) *m/z*: 254 [M+1]^+^, 276 [M+Na]^+^, 529 [2M+Na]^+^}. Anal. Calcd. for C_17_H_19_NO: C, 80.60; H, 7.56; N, 5.53. Found: C, 80.72; H, 7.76; N, 5.43.

**(*Z*)-1-([1,1′-Biphenyl]-4-yl)-*N*-benzylmethanimine oxide (BPMN3).** Following the **General method**, [1,1′-biphenyl]-4-carbaldehyde (91 mg, 0.5 mmol) was treated with NaHCO_3_ (126 mg, 1.5 mmol), Na_2_SO_4_ (213 mg, 1.5 mmol), and BnNHOH.HCl (95.8 mg, 0.6 mmol), in dry THF (20 mL), and irradiated for 4 h, to give, after purification (hex:AcOEt, 7:3, v/v), nitrone **BPMN3** (99.3 mg, 69%), as a white solid: mp 157-9 ºC; IR (KBr) ν 3435, 1148 cm^-1^; ^1^H NMR (400 MHz, CDCl_3_) δ 8.30 (d, *J* = 8.5 Hz, 2H, H3, H5), 7.66 (tt, *J*= 10.1, 1.2 Hz, 2H, H2, H6), 7.63 (d, *J*= 7.2 Hz, 2H, H2’, H6’), 7.55-7.35 [m, 9H: H3’, H5’, H4’, CH_2_C_6_*H*_5_, HC=N(O)], 5.09 [s, 2H, N(O)C*H*_2_C_6_H_5_]; ^13^C NMR (101 MHz, CDCl_3_) δ 143.0 (1C, C1), 140.3 (1C, C1´), 134.0 (1C, C4’’), 133.4 (1C, C4), 129.5 (2C, C3/C5), 129.4 (2C, C3’/C5’), 129.2, 129.1, 129.0 (6C: C4’, C1’’, C2”, C6’’, C3’’, C5’’), 127.9 [1C, HC=N(O)], 127.2 (2C, C2/C6/)*, 127.1 (2C, C2´/C6´)*, 71.2 [C_6_H_5_*C*H_2_(N)O]; MS (ESI) *m/z*: 288 [M+1]^+^, 310 [M+Na]^+^, 597 [2M+Na]^+^. Anal. Calcd. for C_20_H_17_NO: C, 83.59; H, 5.96; N, 4.87. Found: C, 83.34; H, 5.99; N, 4.89.

**General method for the synthesis of bis-nitrones 4-6**. To a solution of [1,1′-biphenyl]-4,4′-dicarbaldehyde (1 mmol) in dry THF (0.05M), Na_2_SO_4_ (4 mmol), anhydrous NaHCO_3_ (3 mmol), and the appropriate hydroxylamine hydrochloride (3 mmol), were added, and the mixture was irradiated (90 ºC/ 15 bar) and stirred for the indicated time in each case. When the reaction was complete (TLC analysis), the solvent was removed, and the crude was purified by flash chromatography.

**(1*Z*,1′*Z*)-1,1′-([1,1′-Biphenyl]-4,4′-diyl)bis(*N*-methylmethanimine oxide) (BPHBN4) [23].** Following the **General method**, [1,1′-biphenyl]-4,4′-dicarbaldehyde (1 mmol (210.2 mg, 1 mmol) was treated with NaHCO_3_ (252 mg, 3 mmol), Na_2_SO_4_ (568 mg, 4 mmol), and MeNHOH.HCl (250.5 mg, 3 mmol) in THF (20 mL), and irradiated for 1 h, to give, after purification (DCM:MeOH, 4%), nitrone **BPHBN4** (243.6 mg, 91%), as a white solid: mp > 220 ºC; IR (KBr) ν 3422, 1164 cm^−1^; ^1^H NMR (400 MHz, DMSO-*d_6_*) δ 8.32 (d, *J*= 8.6 Hz, 4H, H3,5, H3’,5’), 7.91 (s, 2 HC=N(O)), 7.83 (d, *J* = 8.6 Hz, 4H, H2,6, H2’,6’), 3.81 (s, 6H, 2 CH_3_); ^13^C NMR (101 MHz, DMSO-*d_6_*) δ 140.4 (2C, C1,C1´), 133.9 [2C, 2 H*C*=N(O)], 131.0 (2C, C4/C4´), 128.8 (4C, C3/C5/C3´/C5´), 126.9 (4C, C2/C6/C2´/C6´), 54.60 (2C, 2xCH_3_); MS (ESI) *m/z*: 269 [M+1]^+^, 291 [M+Na]^+^, 559 [2M+Na]^+^. Anal. Calcd. for C_16_H_16_N_2_O_2_: C, 71.62; H, 6.01; N, 10.44. Found: C, 71.69; H, 6.27; N, 10.33. 

**(1*Z*,1′*Z*)-1,1′-([1,1′-Biphenyl]-4,4′-diyl)bis(*N*-*tert*-butylmethanimine oxide) (BPHBN5)**. Following the **General method**, [1,1′-biphenyl]-4,4′-dicarbaldehyde (105.1 mg, 0.5 mmol) was treated with NaHCO_3_ (252 mg, 3 mmol), Na_2_SO_4_ (568 mg, 4 mmol), and *tert*-BuNHOH.HCl (376.8 mg, 3 mmol), in THF (20 mL), and irradiated for 8 h, to give, after purification (hex:AcOEt, 1:1, v/v), nitrone **BPHBN5** (108.6 mg, 62%), as a white solid: mp > 220 ºC; IR (KBr) ν 3435, 1361, 1125 cm^−1^; ^1^H NMR (400 MHz, CDCl_3_) δ 8.37 (d, *J*= 8.1 Hz, 4H, H3,5/H3’,5’), 7.71 (d, *J*= 8.1 Hz, 4H, H2,6/H2’,6’), 7.60 [s, 2H, 2 HC=N(O)], 1.64 [s, 18H, 2 x C(CH_3_)_3_]; ^13^C NMR (101 MHz, CDCl_3_) δ 141.6 (2C, C1/C1’), 130.6 (2C, C4/C4’), 129.5 [2C, 2 H*C*=N(O)], 129.5 (4C, C3/C5/C3´/C5´), 127.1 (4C, C2/C6/C2´/C6´), 71.0 [2C, 2 x N(O)*C*(CH_3_)_3_], 28.49 [6C, 2xC(*C*H_3_)_3_]; MS (ESI) *m/z*: 353 [M+1]^+^, 375 [M+Na]^+^, 727 [2M+Na]^+^}. Anal. Calcd. for C_22_H_28_N_2_O_2_: C, 74.97; H, 8.01; N, 7.95. Found: C, 74.60; H, 8.44; N, 7.57. 

**(1*Z*,1′*Z*)-1,1′-([1,1′-Biphenyl]-4,4′-diyl)bis(*N*-benzylmethanimine oxide) (BPHBN6).** Following the **General method**, [1,1′-biphenyl]-4,4′-dicarbaldehyde (210.2 mg, 1 mmol) was treated with NaHCO_3_ (252 mg, 3 mmol), Na_2_SO_4_ (568 mg, 4 mmol) and BnNHOH.HCl (478.8 mg, 3 mmol), in THF (20 mL), and irradiated for 3 h 30 min, to give, after purification (hex:AcOEt, 3:2, v/v), nitrone **BPHBN6** (326.8 mg, 78%), as a white solid: mp > 220 ºC; IR (KBr) ν 3444, 1151 cm^−1^. Anal. Calcd. for C_28_H_24_N_2_O_2_: C, 79.98; H, 5.75; N, 6.66. Found: C, 79.64; H, 6.09; N, 6.59.

### 3.2. Neuroprotection Methods

**Neuroblastoma cell cultures**. The human neuroblastomas cell line SH-SY5Y were cultured in Dulbecco’s: Ham’s F12, 1:1 [vol/vol] containing 3.15 mg/mL glucose, 2.5 mM Glutamax and 0.5mM sodium pyruvate DMEM/F-12, GlutaMAX™; GIBCO, Life Technologies, Madrid (Spain), 1% antibiotic–antimitotic (Gibco; Life Technologies, Madrid, Spain) (containing 100 ui/mL penicillin, 100 mg/mL streptomycin, and 0.25 mg amphotericin B), 1% gentamicin 15 mg/mL (Sigma-Aldrich, Madrid, España) and 10% Fetal Calf Serum (FCS) (Gibco; Life Technologies, Madrid, Spain) as described [27]. Cultures were seeded into flasks containing supplemented medium and maintained at 37 °C in a humidified atmosphere of 5% CO_2_ and 95% air. Culture media were changed every 2 d. Cells were subcultured after partial digestion with 0.25% trypsin-EDTA. For assays, SHSY5Y cells were subcultured in 96 or 48-well plates at a seeding density of 0.50–1 or 2–2.5×10^5^ or cells per well, respectively. When the SHSY5Y cells reached 80% confluence, the medium was replaced with fresh medium containing 0.01–1000 μM compound concentrations or PBS in the controls, as indicated in each assay.

**Neuroblastoma cell cultures exposure to oxygen–glucose deprivation (OGD).** Neuroblastoma cell cultures were exposed to OGD so as to induce cellular damage (experimental ischemia). Cultured cells were washed and placed in glucose-free Dulbecco’s medium (bubbled with 95% N_2_/5% CO_2_ for 30 min) and maintained in an anaerobic chamber containing a gas mixture of 95% N2/5% CO_2_ and humidified at 37 °C at a constant pressure of 0.15 bar. Cells were exposed to OGD for a period of 4 h (OGD 4 h), as indicated. At the end of the OGD period, culture medium was replaced with oxygenated serum-free medium, and cells were placed and maintained in the normoxic incubator for 24 h to recovery (R24h). In the neuroprotection experiments, HBNs and HTNs and PBN (0.01 μM−1 mM) were added at the beginning of recovery period (see below). Control cultures in Dulbecco’s medium containing glucose were kept in the normoxic incubator for the same period of time as the OGD (C4h); then, the culture medium was replaced with fresh medium, and cells were returned to the normoxic incubator until the end of the recovery period (C24h). Control experiments included the same amounts of vehicle (final concentration < 0.01% dimethyl sulfoxide). The experimental procedures were blindly performed, assigning a random order to each assayed nitrone. Nitrones were analyzed independently three to five times with different batches of cultures, and each experiment was run in triplicate.

**Assessment of cell viability.** Measurements of cell viability in human SHSY5Y neuroblastoma cells were carried out in 96-well culture plates as described [22,26]. Briefly, control and treated SH-SY5Y neuroblastoma cells (about 0.75–1×105 cells/well) were incubated with the XTT solution (Cell Proliferation Kit II (XTT), Sigma, Aldrich, Madrid) at 0.3 mg/mL final concentration for 2 h in a humidified incubator at 37 °C with 5% CO_2_ and 95% air (v/v), and the soluble orange formazan dye formed was spectrophotometrically quantified, using a Biotek Power- Wave XS spectrophotometer microplate-reader at 450 nm (reference 650 nm). All XTT assays were performed in triplicate in cells of at least three different cell batches. Control cells treated with DMEM alone were regarded as 100% viability. Controls containing different DMSO concentrations (0.001–1% DMSO) were performed in all assays.

**Measurement of LDH Activity**. For these assays, cultured neuroblastoma cells grown in 96-well culture dishes at a density of 1.5 × 105 cells/well were used. LDH activity was measured as the rate of decrease of the absorbance at 340 nm, resulting from the oxidation of NADH to NAD+ as described [22,28]. Data are given as the percentage of LDH release with respect to the total LDH content (LDH in the culture medium and LDH inside the cells).

**Analysis of Caspase-3 Activity**. For these assays, cultured neuroblastoma cells grown in 48-well culture dishes, at a density of 2.5 × 10^5^ cells/well, were used. After OGD treatment, cells were treated with different nitrones or indicated positive controls at 1−500 μM concentrations and subjected to 24 h reperfusion. Attached cells were lysed at 4 °C in a lysis medium containing 5 mM Tris/HCl (pH 8.0), 20 mM ethylenediaminetetraacetic acid, and 0.5% Triton X-100 and centrifuged at 13.000g for 10 min. The activity of caspase-3 was measured using the fluorogenic substrate peptide DEVD-AMC (66081; BD Biosciences PharMingen), as described [22,29]. Proteins were measured by the Bradford assay. Results were expressed as arbitrary fluorescence units ((AFU)/μg protein/h).

**Measurement of ROS Formation.** SHSY5Y human neuroblastoma cells (2 × 10^5^ cells/well) were exposed to OGD for a period of 4 h (OGD4h). At the end of the OGD period, the culture medium was replaced with oxygenated Dulbecco’s modified Eagle’s medium containing glucose and 10% fetal calf serum. Cells were treated in the absence (controls) or presence of indicated concentrations of nitrones or different known neuroprotective agents and maintained at 37 °C in a normoxic incubator for 3 h for recovery. At the end of this period, 20 μM DHE (HEt; Molecular Probes) was added, and fluorescence was recorded every 15−30 s during a 15 min period, using an excitation filter of 535 nm and an emission filter of 635 nm in a spectrofluorimeter (Bio-Tek FL 600) as previously described [22.29]. Linear regression of fluorescence data (expressed as arbitrary fluorescence units (AFU)) was calculated for each condition, and the slopes (a) of the best fitting lines (y = ax) were considered as an index of O_2_^•−^ production. SNP was used as a positive control of superoxide production [29].

**Statistical Analysis.** Data were expressed as mean ± SEM of results obtained from at least three independent experiments from different cultures, each of which was performed in triplicate. Statistical comparisons between the different experimental conditions were performed using one-way analysis of variance (ANOVA), followed by Holm−Sidak’s post-test when the analysis of variance was significant. A P value <0.05 was considered statistically significant. Fit curves for EC_50_ determinations were performed according to the program of SigmaPlot v.11 (Systat Software INC., 2012).

### 3.3. Antioxidant Activity Tests of BPNs 1-5, PBN And Standards

**Materials and Methods.** Nordihydroguaiaretic acid (NDGA), Trolox, 2,2′-azobis(2-amidinopropane) dihydrochloride (AAPH), 2,2′-Azino-bis(3-ethylbenzthiazoline-6-sulfonic acid) (ABTS) Soybean LOX linoleic acid sodium salt were purchased from the Aldrich Chemical Co. Milwaukee, WI, (USA). Phosphate buffer (0.1 M and pH 7.4) was prepared mixing an aqueous KH_2_PO_4_ solution (50 mL, 0.2 M), and an aqueous of NaOH solution (78 mL, 0.1 M); the pH (7.4) was adjusted by adding a solution of KH_2_PO_4_ or NaOH). For the in vitro tests, a Lambda 20 (Perkin–Elmer-PharmaSpec 1700) UV–Vis double beam spectrophotometer was used.

**Inhibition of linoleic acid peroxidation** [30]. For initiating the free radical, 2,2′-azobis(2-amidinopropane) dihydrochloride (AAPH) is used. The final solution in the UV cuvette consisted of ten microliters of the 16 mM linoleate sodium dispersion 0.93 mL of 0.05 M phosphate buffer, pH 7.4, thermostated at 37 °C. Then, 50 μL of 40 mM AAPH solution was added as a free radical initiator at 37°C under air and 10 μL of the tested compounds. Trolox was used as a reference compound. 

**Inhibition of soybean lipoxygenase** [30]. The oxidation of linoleic acid sodium salt results a conjugated diene hydroperoxide. The reaction is monitored at 234 nm. Soybean lipoxygenase inhibition study in vitro. An in vitro study was evaluated as reported previously.^14^ The tested compounds (several concentrations 1–100 µM, from the stock solution 10 mM were used for the determination of IC_50_) dissolved in DMSO were incubated at room temperature with sodium linoleate (0.1 mM) and 0.2 mL of enzyme solution (1/9 x10^-4^ w/v in saline). The conversion of sodium linoleate to 13-hydroperoxylinoleic acid at 234 nm was recorded and compared with the appropriate standard inhibitor NDGA (IC_50_ 0.45 μM and 93% at 100 μM).

**Hydroxyl radicals scavenging activity** [30]. The hydroxyl radicals were produced by the Fe^3+^/ascorbic acid system. EDTA (0.1 mM), Fe ^3+^ (167 μM), DMSO (33 mM) in phosphate buffer (50 mM, pH 7.4), the tested compounds (0.1 mM), and ascorbic acid (10 mM) were mixed in test tubes. The solutions were incubated at 37 °C for 30 min. The reaction was stopped by CCl_3_CO_2_H (17% *w/v*), and the percentage of scavenging activity of the tested compounds for hydroxyl radicals was given. Trolox was used as a reference compound.

**ABTS^+·^-Decolorization assay for antioxidant activity** [30]. In order to produce the ABTS radical cation (ABTS^+•^), ABTS stock solution in water (7 mM) was mixed with potassium persulfate (2.45 mM) and left in the dark at room temperature for 12–16 h before use. The results are recorded after 1 min of the mixing solutions at 734 nm. The results were compared to the appropriate standard inhibitor Trolox.

## 4. Conclusions

In this work, we have described the design, synthesis, antioxidant, and neuroprotective evaluation against oligomycin A/rotenone, in an in vitro oxygen–glucose deprivation ischemia model, in human neuroblastoma SH-SY5Y cells. In addition, we have described both the capacity to recover the metabolic activity of the neuroblastoma cells from damage induced by both inhibitors of the respiratory chain and ischemia/reperfusion conditions and the activity as inhibitors of necrotic and apoptotic cell death of nitrones **1–5** (Figure 1). 

The results obtained with these **PBN**-derived nitrones show that they are more potent than the reference **PBN** (Figure 1), showing quite similar neuroprotection and antioxidant activity as **NAC**. However, in general, they are less potent in their neuroprotective and antioxidant capacity than the previously investigated homo-*bis*-nitrones, such as **HBN6** (Figure 1) [22] or related homo-*tris*-nitrones [26]. The results from this paper emphasize, once again, that the addition of *N-tert*-Bu and *N-*Bn radicals at the nitrone moiety increases the neuroprotective and antioxidant capacity of the designed **BPNs 1**–**5** (Figure 1), as demonstrated in our previous works [22,26], but the addition of an *N*-Me group produces much weaker effects on the resulting **BPN** activities [22,26]. 

Regarding the conclusion from our previous works that "two nitrone groups are better than one", the results of the present work only partially corroborate it. So, while this hypothesis is supported by the anti-necrotic, antiapoptotic effects, and the neuroprotective capacity of *N-tert*-Bu *bis*-nitrone **BPHBN5** with respect to *N-tert*-Bu mononitrone **BPHBN2**, *N-*Me mononitrone **BPMN1** shows better neuroprotective and antioxidant effects than *N-*Me *bis*-nitrone **BPHBN4**. Therefore, we conclude that although as previously described [22,26], the number of nitrone groups improves the neuroprotection profile of the nitrones, this is not the only factor that can affect their activity, as the incorporation of a new phenyl group at C4 in the structure of **PBN**, which is the final effect of the introduction of a second nitrone group at the new phenyl ring at the C4’ position, will depend on the substituent that is being incorporated, improving the neuroprotection profile in the case of the introduction of a second *N-tert*-Bu group, but not in the case of a second *N-*Me substituent. Finally, note that **BPMN3** substituted with only one *N*-Bn radical is as good as **BPHBN5** bearing two *N-tert*-Bu groups**.** Thus, it would be expected that **BPHBN6**, bearing two *N*-Bn groups, would have even better neuroprotective properties than its corresponding mononitrone **BPMN3**. Unfortunately, due to its insolubility, these tests could not be carried out to verify this. 

To sum up, we conclude the following: (1) Taken together, our results indicate that **BPNs 1**–**5** have better neuroprotective and antioxidant properties than **PBN** and quite similar ones to **NAC**, which is a well known antioxidant agent; (2) Among the nitrones studied, homo-*bis*-nitrone **BPHBN5**, bearing two *N-tert*-Bu radicals at the nitrone motif, has the best neuroprotective, anti-necrotic, anti-apoptotic, and antioxidant activities, followed by mononitrone **BPMN3**, with one *N-*Bn radical, and **BPMN2**, with only one *N-tert*-Bu substituent; and (3) **BPNs** bearing *N-tert*-Bu and *N*-Bn groups show better neuroprotective and antioxidant properties than those substituted with Me.

## Figures and Tables

**Table 1:**
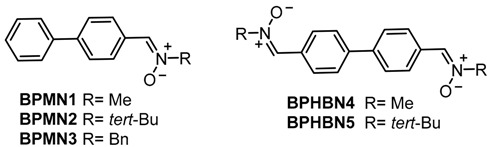
Antioxidant activity of **PBN**, **BPNs 1–5**, **Trolox,** and nordihydroguaiaretic acid (**NDGA**).

Nitrones/Standards	ILPO^a^ (%)	LOX Inhibition (IC_50_ [μM]/ %) ^a^	Scaveging Activity^a^ for ^•^OH (%)	ABTS^+•a^ (%)
PBN	11	23	No	5
BPMN1	No	100 µM	93	4.4
BPMN2	46.5	27	88	No
BPMN3	100	57.5 µM	84	No
BPHBN4	26.5	42	93	2
BPHBN5	68	100 µM	82	No
NDGA		0.45 µM		
Trolox	88		83	91

^a^ Nitrones were tested at 100 µM. Values are means of three or four different determinations. No, no activity under the experimental conditions. Means within each column differ significantly (*p* < 0.05).

## Data Availability

Not applicable.
